# Does the right temporo-parietal junction play a role in processing indirect speech acts? A transcranial magnetic stimulation study

**DOI:** 10.1016/j.neuropsychologia.2023.108588

**Published:** 2023-09-09

**Authors:** Isabella P. Boux, Friedemann Pulvermüller

**Affiliations:** a Brain Language Laboratory, Department of Philosophy and Humanities, WE4, Freie Universität Berlin, Habelschwerdter Allee 45, 14195, Berlin, Germany; b Einstein Center for Neurosciences, Charitéplatz 1, 10117, Berlin, Germany; c Berlin School of Mind and Brain, Humboldt Universität zu Berlin, Luisenstraße 56, 10117, Berlin, Germany; d Cluster of Excellence ‘Matters of Activity. Image Space Material’, Humboldt Universität zu Berlin, Unter Den Linden 6, 10099, Berlin, Germany

**Keywords:** Indirect speech acts, Theory of mind, Right temporo-parietal junction, Transcranial magnetic stimulation, Neuropragmatics

## Abstract

In communication, much information is conveyed not explicitly but rather covertly, based on shared assumptions and common knowledge. For instance, when asked *“Did you bring your cat to the vet?”* a person could reply *“It got hurt jumping down the table”*, thereby implicating that, indeed, the cat was brought to the vet. The assumption that getting hurt jumping down a table motivates a vet visit is tacitly attributed to the speaker by the listener, which implies Theory of Mind (ToM) processes. In the present study, we apply repetitive transcranial magnetic stimulation to the right temporo-parietal junction (rTPJ), a key brain region underlying ToM, with the aim to disrupt ToM processes necessary for language understanding. We then assess effects on the comprehension of indirect speech acts and their matched direct controls. In one set of conditions, the direct and indirect stimuli where not matched for speech act type, whereas, in the other, these were matched, therefore providing an unconfounded test case for in/directness. When indirect speech acts and direct controls were matched for speech act type (both *statements*), the indirect ones took longer to process both following sham and verum TMS. However, when the indirect and direct speech acts were not matched for communicative function (*accept/decline* offer vs. descriptive *statement* respectively), then a delay was detected for the indirect ones following sham TMS but, crucially, not following verum TMS. Additionally, TMS affected behavior in a ToM task. We therefore do not find evidence that the rTPJ is causally involved in comprehending of indirectness *per se*, but conclude that it could be involved instead in the processing of specific social communicative activity of *rejecting* of *accepting* offers, or to a combination of differing in/directness and communicative function. Our findings are consistent with the view that ToM processing in rTPJ is more important and/or more pronounced for offer *acceptance/rejection* than for descriptive answers.

## 1 Introduction

### 1.1 Linguistic indirectness

Linguistic indirectness is a common phenomenon in human communication. When a person asks *“Did you bring your cat to the vet?*” and the hearer replies *“It got hurt jumping down the table.”* it is usually clear that the hearer is thereby confirming that he/she is bringing the cat to the veterinary, in spite of the absence of an explicit statement to this end. This reply is an example of an indirect speech act, because the speaker *“utters a sentence, means what he says, but also means something more”* (Searle, 1979). According to Grice, indirect speech acts can be understood by the hearer because they assume that the so-called Maxim of Relevance is obeyed, that is, that the speaker says something of relevance for the current scopes of the ongoing conversational exchange (Grice, 1975). This linguistic phenomenon is therefore also known as a Relevance implicature. According to the Gricean and Searlean framework, it is then up to the speaker, to deploy a chain of inferences allowing them to identify the intended communicated message. This inferential chain can be worked out by relying on the assumption of relevance, combined with world knowledge, contextual information and rationality. In particular, Theory of Mind (ToM), the capacity to ascribe mental states and processes to others, has been thought to play a central role in comprehension of indirectness, above and beyond what is usually involved in any communicative situation. Namely, it has been thought to contribute to understanding the communicative intention conveyed indirectly by the speaker. When contextualizing the phenomenon of indirect speech acts in the broader picture of pragmatic phenomena, two main positions have emerged. On one hand it has been argued that pragmatic processing is achieved by a dedicated sub-module of a more general ToM module (Sperber and Wilson, 1995, 2002), predicting ToM involvements in all cases of pragmatic processing. Recently, however other positions have emerged which attempt to scale back the role of ToM in pragmatic processing. First, some have criticized that the measures used in the past to independently asses ToM function (such as in Happé, 1994) partially conflate ToM with pragmatics function and therefore lead to an overestimation of the relationship between ToM processing and pragmatic processing (Bosco et al., 2018). Additionally, others have suggested that ToM might come into play only for certain pragmatic phenomena, but not all (Andrés-Roqueta and Katsos, 2017; Bosco et al., 2018; Domaneschi and Bambini, 2020; Jary, 2013; Kissine, 2016). Only processing of certain pragmatic phenomena which clearly are about inferring about the speaker's mental state (e.g., irony) would require ToM, while other phenomena might rather hinge on linguistic knowledge, general world knowledge or possibly non-ToM inferencing. Investigating ToM involvement to comprehension of indirect speech acts can therefore provide new insights contributing to this broader question.

### 1.2 Indirect speech acts and speech act type

Several studies have been conducted on the neurocognitive processes of understanding of indirect speech (see Section 1.3 for a review). Investigating indirectness involves comparing utterances that are used to carry out indirect speech acts to utterances used to carry out direct ones. This strategy however also comes with a risk. Sentences used in direct and indirect speech act conditions differ in structural dimensions such as lexical, morphological or syntactic ones. One solution to avoid these putative problems is to employ identical sentences and utterances for direct and indirect speech act conditions, which may minimize the likelihood of these confounds. This approach has been largely adopted in the last years. For instance, van Ackeren et al. (2012) used exactly the same utterance “*It is hot here*” in direct and indirect speech act conditions. In the context of a desert landscape, the utterance was only used to *describe* the temperature in the desert. However, in the context of the picture of a room with a closed window, it could be understood as carrying out an indirect speech act, specifically an indirect *request*. Although using this approach successfully achieves a structural comparability between the direct and indirect stimuli, it does not achieve functional comparability. Indeed, the two experimental conditions differed not only in their indirectness, but also in the *type* of speech act (SA, i.e., communicative function, illocutionary act/role) that they carried out, namely a *description* and *request*. Therefore, the necessary precaution of using identical sentence does not guarantee successful matching of direct and indirect speech act conditions. Any difference in behavior or brain activity between the conditions of van Ackeren et al. (2012) could, in fact, be a result of their difference in/directness, of that in speech act type, or both. The communicative function of the speech act needs to be taken into account and excluded as a possible confound when investigating this phenomenon. We note however that – to the best of our knowledge - all previous studies of indirect speech acts were subject to this or the previous confounds regarding utterance form and/or speech act function.

This consideration is of relevance as different (direct) speech acts have been found to be associated with varying patterns of activations in the brain. For instance, a range of neuroscientific methods, including EEG, fMRI and MEG allowed to compare *request* with *naming* SAs. It was shown that comprehending or producing *requests* conveyed by same utterances is associated with greater activations of the motor system (Boux et al., 2021; Egorova et al., 2013, 2014, 2016; Tomasello et al., 2019; see also Tomasello et al., 2022) and also of ToM regions such as the rTPJ along with Broca's area and bilateral parietal and temporo-occipital areas (Egorova et al., 2013, 2016) compared with comprehension of the same utterances used to perform a *naming* speech act. Interestingly, when *requests* are performed indirectly (e.g., “*It is warm in here*” as an indirect *request*to open the window), they are also associated with BOLD activations in the action system (van Ackeren et al., 2012, 2016), similar to direct requests. It therefore appears that brain signatures specific to certain speech act types might be present also when these are performed indirectly. In other words, it is possible that additional cognitive mechanisms are required for the listener to process the speech act type performed indirectly, when contrasted with the same utterance performing a different speech act directly.

### 1.3 Processing of indirect speech acts

Examining the literature shows that the typical approach used so far – both in behavioral and neuroscientific studies - has been to contrast a given utterance used to perform a certain type of direct speech act with the same utterance used to indirectly perform a different speech act. Such a contrast however may, together with indirectness, also capture neurocognitive signatures of speech act type processing that are not inherent to indirectness processing *per se*.

First, behavioral studies consistently reported that indirect speech acts take longer to be understood than direct ones (Jang et al., 2013; Shibata et al., 2011). Importantly, this difference persists also when direct and indirect speech acts performed by the means of the same utterance are compared (Feng et al., 2017, 2021; Hamblin and Gibbs, 2003; Holtgraves, 1999). These results indicate that additional processing costs are required to understand a given utterance when it is intended as an indirect rather than as a direct speech act. These processing delays are often attributed to the process of inference that, in the Searle-Grice perspective, the receiver must engage in to understand the indirectly conveyed message. The Searlean process of comprehending indirect speech acts is based on a multi-step process involving, among others, understanding the direct meaning of the utterance, detecting that the speaker is deviating from general cooperative principles of conversation, identifying that the deviation is intentional and then using general world knowledge that is part of the common ground as well as deductive reasoning to access the implicated meaning. While this multi-step procedure requiring *explicit* reasoning may appear not fully plausible (Searle, 1979), understanding of indirect speech acts might require an increased engagement of the cognitive function of Theory of Mind (ToM) processing to monitor the common ground including shared assumptions and intentions (Stalnaker, 2002). Although communication in general is likely to require ToM processing, it appears plausible that indirect speech acts are more strongly dependent on it, as additional inferential processes may be engaged to determine the implicated propositional content and communicative function. Indeed, some studies find a link between comprehension of indirect *requests* and ToM in healthy adults (Trott and Bergen, 2018) and clinical populations (Champagne-Lavau and Joanette, 2009; Champagne-Lavau and Stip, 2010). For instance, individual differences in ToM abilities predicted how likely healthy subjects were to interpret a remark as an indirect *request* (Trott and Bergen, 2018). Similarly, it was found that the accuracy in a meta-linguistic indirect *request* comprehension task was predicted by ToM function in right hemisphere damaged patients (Champagne-Lavau and Joanette, 2009) and in schizophrenic patients (Champagne-Lavau and Stip, 2010). Importantly, however, altogether these behavioral studies did not report whether the speech act type carried out in the direct and indirect conditions were matched (Feng et al., 2017, 2021; Hamblin and Gibbs, 2003; Holtgraves, 1999) or deliberately compared direct non-directive speech acts to indirect directives (Champagne-Lavau and Joanette, 2009; Champagne-Lavau and Stip, 2010). Therefore, in these studies, the confound related to speech act type was either present, or it could not be ruled out.

From a neural perspective, ToM function has been known to be consistently associated with a set of brain areas. These include the temporo-parietal junction (TPJ), the medial prefrontal cortex (mPFC), the precuneus (PreCu) and – although less consistently - the temporal poles (TP) and the posterior superior temporal sulci (pSTS) (Schurz et al., 2013, 2014, 2021; Van Overwalle and Baetens, 2009). Functional magnetic resonance (fMRI) studies have assessed which neural networks are active during comprehension of indirectness in healthy neurotypical adults. These studies have consistently found that the ToM brain network – particularly the rTPJ and the mPFC - is more strongly activated for indirect replies vs. direct replies (Jang et al., 2013; Shibata et al., 2011). This finding was reported also when direct and indirect replies were conveyed by the same utterance (Bašnáková et al., 2014, Bašnáková et al., 2015, Feng et al., 2017, Feng et al., 2021, van Ackeren et al., 2012, van Ackeren et al., 2016). Also in this neuroimaging literature, however, it was not the case or was not clear whether indirect speech acts were compared with direct ones conveying same communicative function. For instance, a recent study addressed the speech act type confound by comparing direct speech acts with indirect *requests* and, in addition, with non-*request* indirect “replies” conveyed by the same utterance. For example, the sentence “*It is quite far away*” was used as a *statement* directly answering a question (“*How far away is China?“),* as a *request* indirectly responding to an offer (“*Shall I move the TV closer?*“) and, as the newly added control condition, an “indirect answer” to a question (“*Have you started preparing for the exam?*“). However, also in this latter condition, no mention is made of SA-matching. Indeed, the reported example stimuli show that the “indirect reply” functions, in addition, as an *excuse*. These authors claimed that the ToM network activation patterns observed during comprehension of indirect requests are to be attributed to indirectness rather than to the fact that a *request* is being expressed (van Ackeren et al., 2012, 2016). This however omits the possible role of the different illocutionary roles of their direct and indirect replies. Therefore, overall, neuroimaging studies investigating the neural signatures of indirectness do consistently find associations between stronger activations in the ToM system and comprehension of indirectness. However, the interpretability of these neuroimaging results in unclear, given that the confound of speech act type was not sufficiently controlled for.

Additionally, neuroimaging studies only allow for identifying correlations between a given cognitive process and activations in specific brain areas. So far, only one study attempted to address the issue of causality of the ToM-supporting rTPJ region in processing indirectness (Feng et al., 2021). They directed transcranial direct current stimulation (tDCS) to the right temporo-parietal junction (rTPJ), which, as mentioned above, is important for ToM processing (Saxe and Wexler, 2005; Schurz et al., 2014). They subsequently observed alterations in the performance in an indirect SA comprehension task and an alteration of performance in a ToM task. An additional analysis indicated that the alterations in the comprehension of indirect speech acts were mediated by ToM function. However, similar to previously discussed studies, no speech act matching appeared to have been applied.

So, overall previous studies on indirectness did not isolate the phenomenon of indirectness form other factors, in particular an additional, potentially confounding difference in SA function, as they did not choose stimuli in which the same utterances were used to perform the same type of speech act both in the direct and indirect conditions. Additionally, among the indirect speech acts, a predominance of speech acts known to be associated with ToM (e.g., *requests*) or likely to be so (e.g., *excuses*, *promises*, *negative opinions*, etc.) can be noticed. It therefore cannot be excluded that the increased ToM activations found for comprehension of indirectness were in fact due to processing these specific types of speech acts rather than indirectness *per se*. On the background of this body of research, it is necessary to re-evaluate the evidence on indirectness of speech acts with different communicative functions in order to find out whether previous findings were due to indirectness or rather to any differences in the communicative function (i.e., speech act type, illocutionary force/role) of the investigated utterances. Whether ToM brain regions are causally involved in processing indirect speech acts is therefore still unclear. We here focus on one ToM network region: the rTPJ. This region was frequently found to be active in the studies reviewed above, despite the speech act type confound (Bašnáková et al., 2014, 2015; Bendtz et al., 2022; Feng et al., 2017, 2021; van Ackeren et al., 2012). In addition is has also been found to be linked to processing of other pragmatic phenomena, such as metaphors, irony and humor (Bambini et al., 2011; Campbell et al., 2015; Carotenuto et al., 2018; Spotorno et al., 2012) although with moderate consistency.

### 1.4 Experimental design and predictions

Here we ask whether the rTPJ is causally involved in processing indirect speech acts. Following the behavioral rating study by Boux et al. (2023), we report the first neurolinguistic study comparing the processing of direct and indirect speech acts that were performed with identical utterances and were matched as closely as possible for their illocutionary role or communicative function. In addition, a second set of stimuli was used, where direct and indirect speech acts were performed with the same utterance but had different speech act functions, similar to the approach used in previous research. Subjects were delivered verum repetitive TMS to the right TPJ in one condition, and sham stimulation in another condition. Following verum or sham TMS, subjects underwent a pragmatic task where they were shown direct and indirect replies. They were asked to judge whether the replies could be understood as “yes” or “no”. Response times were measured and evaluated against the following predictions: (i) response times are longer for indirect than for direct speech acts; (ii) TMS to the rTPJ alters reaction time differences between direct and indirect speech acts. A set of alternative predictions postulated that processing differences between direct and indirect speech acts are altered for stimuli matched for communicative function type. In this case, predictions (i) and (ii) would hold for unmatched, i.e., speech-act confounded conditions, but no for matched ones. As the behavioral modulation following rTPJ stimulation is frequently attributed to a modulation of ToM processing, we also had our subjects perform a ToM task to ascertain that ToM processing was indeed affected by TMS.

## 2 Material and methods

All tasks were programmed in Matlab (2012b, The MathWorks Inc., Natick, MA) in combination with the Psychtoolbox 3 toolbox. Statistical analyses were conducted with Python 3.6 and R (RC-Team, 2019).

### 2.1 Experimental subjects

28 subjects were tested for the present study. Subjects were included if they (i) were aged between 18 at 35 years, (ii) were right-handed, (iii) did not wear any implant that was incompatible with TMS, (iv) were native speakers of English and grew up in a monolingual environment (v) had no neurological or psychological disorder and (vi) were not color-blind. The study was carried out in accordance with the Ethics Committee of the Charité Universitätsmedizin (Berlin, Germany), which approved all the study procedures.

Of the 28 tested subjects, three underwent the first testing session only and therefore had data either in the sham or verum condition. One subject was fully excluded from the analysis because of not meeting the inclusion criteria of right-handedness based on the Edinburgh Handedness Test (Oldfield, 1971). One was excluded in the pragmatic task analysis and one more in the ToM Task analysis as they appeared not to have understood the task in one of the two testing sessions. After subject exclusion our final sample size was of 27 subjects in total (14 females, 13 males), of which 26 in the pragmatic and 26 in the ToM task. Our final sample had a mean age of 23.7 years ±4.4 SD. In addition, the included subjects had a mean LQ of 80.5 ± 24.1 SD as assessed by the Edinburgh Handedness Test, which confirmed their right-handedness (Oldfield, 1971). All participants signed an informed consent form prior to the beginning of the experiment and received a monetary compensation.

### 2.2 Pragmatic task

The pragmatic task was based to a large extent on the same stimuli used by Boux et al. (2023). It consisted of critical replies that were presented in two alternative contexts consisting of interrogative sentences. One context favored a direct reading of the critical reply (direct condition), while the other favored an indirect reading (indirect condition). Therefore, the critical stimulus, namely the reply to the context sentence, was identical for direct and indirect condition (see Table 1). Because the context sentences were always polar questions, the critical replies were always interpretable as conveying a “yes” or a “no”. Critical replies were always expected to be interpreted in the same way in both their contexts (e.g., always either as a “yes” or as a “no”). In addition, there were two sets of stimuli. In the Speech-Act-matched set (SA-matched), the context sentences both in the direct and indirect conditions were used to perform what Searle classifies as “true” question (i.e., querying information) and the replies were *statements* providing that information. In the non-Speech-Act-matched (non-SA-matched) condition, the interrogative sentence constituting the direct context was still querying information, so that the direct reply would be providing this information. However, the same context sentence in the indirect condition was an offer or proposal, so that the indirect reply was in fact the *refusal*or *acceptance*of the offer. The SA-matched set included 70 pairs of direct/indirect items, whereas the non-SA-matched set included 62. In each set, half of the pairs were to be interpreted as “yes” and half as “no”. Both our a priori classification of the interpretation as a “yes” or a “no” and the in/directness of the critical stimuli were validated by an independent sample of 28 participants (Boux et al., 2023). Indirect replies were rated as significantly less direct than their direct counterpart. Before the experiment, subjects were informed that they would be shown question-reply pairs, drawn from conversation between people in which one person asks the question and the other replies. It was specified that the reply was a complete conversational turn (i.e., that the person replying was not adding further utterances after the reply itself). Subjects were instructed to indicate as quickly and accurately as possible by pressing a key with their right hand whether they interpreted the reply as a “yes” or “no”.

**Table 1:** Examples of stimulus material in the SA-matched and non-SA-matched conditions with direct and indirect speech acts. For each condition, the communicative function of the interrogative sentence constituting the context and of the critical reply are given in columns headed CONTEXT SA and REPLY’S SA, respectively. The last column indicates whether the reply is meant to be interpreted as a “yes” or “no” to the context question.

SET	CONDITION	CONTEXT SENTENCE	CONTEXT SA	CRITICAL REPLY	REPLY’S SA	EXPECTED INTERPRETATION
SA-matched	direct	**Is your cat hurt?**	true question	**It got wounded.**	true answer	yes

	indirect	**Are you bringing your cat to the vet?**	true question	true answer	yes

direct	**Does Megan eat meat?**	true question	**She is vegetarian.**	true answer	no

indirect	**Is Megan coming to the steakhouse?**	true question	true answer	no

non-SA-matched	direct	**Are you eating something?**	true question	**I am having a soup.**	true answer	yes
	indirect	**Do you want a spoon?**	offer (or proposal)		accepting the offer	yes

	direct	**Have you decided on a destination?**	true question	**We are not sure where to go yet.**	true answer	no
indirect	**Shall I buy the train tickets?**	offer (or proposal)	rejecting the offer		no

To exclude potential context effects due to similarity between context questions and critical replies in the direct and indirect conditions, these conditions were matched for a set of psycholinguistic properties: length of the context question counted in words, pronoun repetitions between context sentence and critical reply, number of coreferences between the context sentence and the critical reply, number of repeated lemmas between context and critical reply as well as semantic similarity between context question and reply sentences, calculated as the cosine between their semantic vectors obtained based on Latent Semantic Analysis (LSA, Landauer et al., 1998, Landauer et al., 2007). LSA is a method that allows to generate multi-dimensional semantic spaces based on word co-occurrences within documents in a corpus. Individual words can then be represented in this semantic space as vectors. Even entire (novel) sentences can be represented as vectors resulting from the sum of the vectors of their individual word components. Therefore, the semantic distance between two sentences can be calculated as the cosine of the angle between two vectors. The cosine similarity between each question and its corresponding reply was obtained from the online tool, http://lsa.colorado.edu/, selecting the term-to-term comparison and applying it to the tasaALL semantic space (300 semantic dimensions). The corpus on which the semantic space was based included texts from different sources such as novels, newspaper articles and other texts, which were estimated to correspond to the reading level up to a first-year college student.

All above mentioned properties were matched between the eight conditions resulting from the crossing of the factors of SA-matching, reply's expected Interpretation and In/directness (see Table 2 and Table 3). Specifically, differences in cosine similarity and length of context question between conditions were not significant as assessed by a 2 × 2 × 2 ANOVA with the factors In/directness [direct, indirect], SA-matching [SA-matched, non-SA-matched] and Interpretation [yes, no] (all main and interaction effects had *p* > 0.05, Table 3). Number of repeated pronouns, number of coreferences and number of repeated lemmas were also comparable between conditions, as assessed by likelihood-ratio chi-squared tests applied to all (12) relevant pairwise comparisons (all *p* > 0.05, see Table 3). Finally, we also tested differences in length of critical reply and in number of content words in the critical replies by the means of a 2 × 2 ANOVA with factors SA-matching [SA-matched, non-SA-matched] and Interpretation [yes, no]. These were also not statistically significant (all main effects and interaction effects had *p* > 0.05, see Table 2). All stimuli were divided in two matched lists, so that for each pair, the direct and indirect version of the stimulus were on separate lists. Because each subject performed the task twice (once in the verum session and once in the sham session), one list was used for each session and the attribution of a list to the first or second experimental session was counterbalanced across subjects. Therefore, the same critical reply was presented twice to each subject, but in separate sessions: once in the direct condition and once in the indirect condition.

**Table 2:** Psycholingustic properties of the stimulus material. The items are split by SA-matching (SA-matched, non-SA-matched), Interpretation (Yes, No) and In/Directness (Direct, Indirect). The number of items (n) is indicated for each condition. Note that SA-matched and non-SA-matched differ in their number of items. None of the measures significantly differed across conditions.

	SA-matched (n = 70)		non-SA-matched (n = 62)	
	Yes (n = 35)	No (n = 35)	Yes (n = 31)	No (n = 31)
**Length critical utterance in words (mean** ± **SD)**	5.46 ±1.54	5.23 ±1.21	5.90 ±1.25	5.52 ±1.46
**Number of content words in critical utterance (mean** ± **SD)**	2.77 ±1,03	2.71 ±0.62	2.81 ±0.95	2.55 ±0.85

**Table 3:** 2.4 Psycholingustic properties defining the relationship between the critical replies and their context question. The items are split by SA-matching (SA-matched, non-SA-matched), Interpretation (Yes, No) and In/Directness (Direct, Indirect). The number of items (n) is indicated for each condition. Note that SA-matched and non-SA-matched differ in their number of items. None of the measures significantly differed across conditions.

	SA-matched (n = 140)				non-SA-matched (n = 124)			
	Yes (n = 70)		No (n = 70)		Yes (n = 62)		No (n = 62)	
	Direct (n = 35)	Indirect (n = 35)	Direct (n = 35)	Indirect (n = 35)	Direct (n = 31)	Indirect (n = 31)	Direct (n = 31)	Indirect (n = 31)
**Cosine similarity (mean** ± **SD)**	0.68 ±0.13	0.65 ±0.17	0.65 ±0.15	0.65 ±0.13	0.68 ±0.11	0.65 ±0.13	0.64 ±0.12	0.62 ±0.11
**Length context question in words (mean** ± **SD)**	5.66 ±1.21	5.54 ±1.36	6.06 ±1.33	6.29 ±1.27	5.97 ±1.58	6.16 ±1.16	6.06 ±1.31	5.94 ±1.29
**Number of repeated pronouns (sum)**	8	8	6	6	4	2	1	3
**Number of coreferences (sum)**	32	32	35	35	34	32	36	36
**Number of repeated lemmas (sum)**	9	12	11	8	8	7	7	8

The stimuli were presented as black text appearing in the center of the screen on a grey background. First the context sentence was presented on screen for 2 s, then disappeared and was followed by a fixation cross for 0.5 s. Then the critical reply appeared and remained on screen for 3 s during which the subject had to respond, and response times were recorded. Finally, the critical reply disappeared, and the trial was concluded with a fixation cross appearing for 2 s, before the next trial started. Responses were given via a left or right arrow key press. The correspondence between key and interpretation as "yes" or "no" was randomized across subjects but kept constant across sessions. Each session contained 132 trials and lasted about 17 min.

### 2.3 Theory of mind task

The Theory of Mind task was originally designed by Apperly et al. (2011). In the present design we used the improved version developed by Hartwright et al. (2012). The task measures two relevant aspects of Theory of Mind: processing of belief and processing of desire. Experimental subjects needed to predict the behavior of a fictional character based on the character's beliefs (true belief: B+, false belief: B-) about the location of a given food item and the character's desire for that item (approach: D+, avoidance: D-). The factors of Belief and Desire were orthogonal and therefore yielded four different conditions.

Each trial started by the presentation of three different types of statements (see Table 4): (i) state of affair statement, indicating whether a given food item *is located* in a red or in a blue box, (ii) belief statement, indicating whether the character *believes* that the food item is in the blue or red box and (iii) desire statement, indicating whether the character *loves* or *hates* the food item. Within a trial, these statements were presented sequentially in randomized order for 1.2 s each and separated by a 0.4s blank screen. Subsequently, a fixation cross was displayed for 0.4s and, and followed by either one of two images presented for 1.7 and during which the experimental subjects were expected to produce a button press. In test trials (66.6% of trials), the image depicted the character sitting at a table with a blue and red box at each side. In such case, the subject was expected to indicate by button press which box the character would open, based on his/her beliefs and desires. In catch trials (33.3% of trials), a similar image was presented, but the character was absent and replaced by a question mark signaling that the experimental subject had to indicate the *real* location of the food item. Finally, in both trial and catch trials, a fixation cross appeared for 3s until the next trial started. The task had 96 trials per session and lasted about 15 min. Subjects were asked to read the statements carefully and, as soon as the image with the character appeared, they had to indicate as quickly and accurately as possible which box (left vs. right) the character would open by pressing the corresponding key. Conversely, in the catch trials, they were instructed to indicate where the food item was actually located instead (irrespective of the character's belief and/or desires).

**Table 4:** Examples of stimuli in the various conditions of the Theory of Mind Task resulting from crossing the factors Belief (B+: true belief, B-: false belief) and Desire (D+: approach, D-: avoidance).

Condition	State of affair statement	Belief statement	Desire statement	Correct response in test trials	Correct response in catch trials
**B + D+**	Donuts are in the **red** box.	He thinks the donuts are in the **red** box.	He **likes** donuts.	red box	red box
**B-D+**	Donuts are in the **red** box.	He thinks the donuts are in the **blue** box.	He **likes** donuts.	blue box	red box
**B + D-**	Donuts are in the **red** box.	He thinks the donuts are in the **red** box.	He **hates** donuts.	blue box	red box
**B-D-**	Donuts are in the **red** box.	He thinks the donuts are in the **blue**box.	He **hates** donuts.	red box	red box

### 2.4 TMS protocol

TMS was delivered using a MagPro X100 system (MagVenture, Farum, Denmark). A 70 mm figure-of-8 coil was used in the verum condition and a sham coil was used in the sham condition. These two coils were indistinguishable to the subjects who therefore were blind to the nature of the sham or verum stimulation. TMS was applied off-line to the rTPJ prior to the pragmatic and Theory of Mind tasks. The stimulation protocol was taken from Young et al. (2010) because of the similarity with our research question and study design. Indeed, Young et al. targeted the rTPJ and successfully induced changes in processing of beliefs in a moral judgement task. The protocol consisted of biphasic pulses at a frequency of 1 Hz applied for 25 min with the handle of the coil pointing backwards. This resulted in a total of 1500 pulses. The only modification that we introduced was the use of the resting motor threshold (RMT) to define the intensity of the stimulation. In the context of RMT procedure, TMS pulses were directed to the right motor cortex and the resulting motor-evoked potential (MEP) were measured by electromyography of their left *Abductor pollicus brevis* (APB) in a belly-tendon montage. The resting motor threshold was defined as the intensity which elicited an MEP response larger than 50 μV in 5 out of 10 pulses in the APB muscle (Rossini et al., 1994). The final stimulation intensity was set to 90% of the RMT, as it is common in TMS research (e.g., see Donaldson et al., 2015). In the few cases where the final stimulation intensity provoked muscle twitches in the subjects (e.g., in proximity of the right eye or the right jaw muscles), the final stimulation intensity was decreased until the twitch disappeared. RMT procedure took place in both in preparation of verum and sham TMS, to ensure similarity between conditions from the participant's perspective. Our stimulation target was determined based on a meta-analysis by Krall et al. (2015), which found that the posterior rTPJ was recruited selectively for false-belief tasks as opposed to the anterior rTPJ which was active both during false-belief and attentional tasks. Therefore, based on Krall et al. (2015) we targeted the peak coordinates in the posterior rTPJ (MNI [x = 54, y = −58, z = 27]) as our stimulation target. The point on the scalp above the stimulation target was localized on each subject's head based on the EEG 10-10 system (Herwig et al., 2003; Okamoto et al., 2004). Using the projection of standard electrodes locations on a Talairach brain template (Koessler et al., 2009) and subsequently converting them to MNI coordinates (on-line tool previously available at: http://sprout022.sprout.yale.edu/mni2tal/mni2tal.html), we determined that the stimulation target was located mid-way between the electrodes P6 and CP6. rTMS is usually considered to alter brain activation for longer than 30 min after termination of stimulation (Hartwigsen, 2015, Valero-Cabré et al., 2007) which should be sufficient to cover both our tasks, which had a joint duration of 32 min. All our subjects wore earplugs during both verum and sham treatments. Our overall stimulation parameters were well within the established safety guidelines (Rossi et al., 2009) and subjects underwent a standardized safety screening questionnaire (Rossi et al., 2011) prior to undergoing any TMS-related protocol.

### 2.5 Experimental procedure

Each subject was invited for two experimental sessions that took place with a 2–3 weeks interval (median of 14 days). On one of the sessions verum TMS was delivered, while on the other sham TMS was delivered. The order of verum and sham TMS was counterbalanced across participants. In the first session, the subjects filled out a demographic questionnaire, and the Edinburgh Handedness Test (Oldfield, 1971). In each session, subjects performed the two computerized tasks. Half of the subjects always started with the pragmatic task, while the other half always started with ToM task. Both tasks were trained immediately prior to the TMS procedure. The training consisted of 10 trials per tasks. The experimenter monitored accuracy and provided additional guidance if needed. The training ensured that participants had understood the task and that they got familiarized with the task's timing. Therefore, overall, in the present study, task order within a session, stimulation order between sessions and attribution of stimuli list to the verum or sham condition in the pragmatic task were counterbalanced across subjects, while response key in the pragmatic task was randomized. Each subject was randomly assigned to each of these “condition combinations”.

### 2.6 Preprocessing

In addition to incorrect responses in both tasks, all trials with reaction times above 3s in the pragmatic Task and above 1.7s in the ToM task were also counted as incorrect. RTs were normalized by log10 to meet the Gaussian distribution assumption required for further statistical analysis. Normalized RTs for incorrect trials or RTs that were more than 2SD away from the condition mean of any given subject (Hartwright et al., 2012) were not analyzed. Therefore, concerning the RT data of the pragmatic task, an average of 5.93 ± 3.01% SD of trials were excluded because subjects responded incorrectly and an additional average of 3.73 ± 0.94% SD were excluded because they were beyond 2SD from the condition log10 (RT) mean. This resulted in an average of 90.34 ± 3.03% SD of trials per subject and 5863 trials in total entering the final RT analysis.

Concerning the RT data from the ToM task, an average of 11.0 ± 6.8% SD of trials were excluded because subjects responded incorrectly, 3.09 ± 1.07% SD were excluded because they were beyond 2SD from the condition mean, thus retaining an average of 85.91% ± 6.75% SD of trials per subject in the final sample. This resulted in a total of 2707 trials entering the final RT analysis.

### 2.7 Statistical evaluation

The accuracy and log-normalized RT data were analyzed in R (RC-Team, 2019) with linear mixed models, as implemented in the *lme4* package (Bates et al., 2015). The function *lmer*() was used for continuous reaction time data whereas the *glmer*() function was used for binary accuracy data. Based on our hypothesis, the model included all our variables of interest as predictor variables. In addition to these, some putative confounds were added to the fixed structure of the model. For the pragmatic task, these were: length of the target sentence in words (centered) and experimental session (first vs. second). For the ToM task only experimental session (first vs. second) was added. Finally, the model included by-subject and by-item intercepts. Sum contrast coding (i.e., [1, −1]) was used for all categorical predictors (In/Direct: direct_[1]_, indirect_[-1]_; SA-matching: SA-matched_[1]_, non-SA-matched_[-1]_; Stimulation: sham_[1]_, verum_[-1]_; Session: first_[1]_, second_[-1]_; Belief: B+_[1]_, B-_[-1]_; Desire: D+_[1]_, D-_[-1]_). The structure of the models is reported below in Wilkinson notation for the Pragmatic (P) and the ToM (T) tasks. The residuals were visually inspected to ensure that they met the assumptions of normality, equivariance and independence. Statistical significance of the predictors was computed based on Satterthwaite's method for estimation of degrees of freedom as implemented in the *lmerTest* package ( Kuznetsova et al., 2017). Post-hoc tests were performed with the *emmeans* () function of the emmeans package (https://cran.r-project.org/web/packages/emmeans/index.html) using the implemented Tukey HSD correction for multiple comparisons.

(P) Variable ∼ In/Directness * Stimulation * SA-matching + length + session + (1|subject) + (1|item).

(T) Variable ∼ Belief * Desire * Stimulation + session + (1|subject)+ (1|item).

## 3 Results

### 3.1 Pragmatic task

**Fig. 1:**
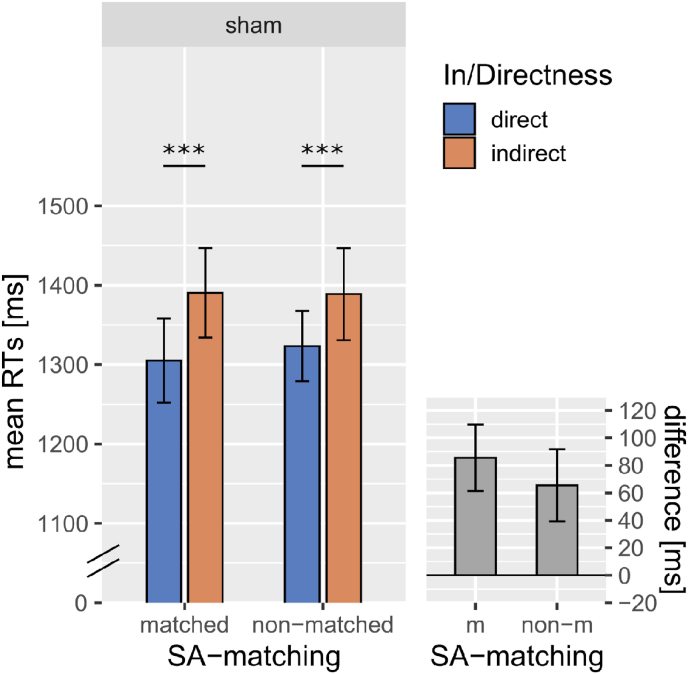
The large panel illustrates the average RTs (by subject) form the pragmatic task in the sham TMS condition plotted by SA-matching and In/Directness, illustrating the significant effect of In/Directness. The small panel illustrates the average difference in RTs found in the sham data between direct and indirect conditions (indirect >direct), separately by SA-matching. Error bars indicate the standard error of the mean (SEM) by subject. * indicates p < 0.05, ** indicates p < 0.01, *** indicates p < 0.001).

Analysis of the reaction time data in the sham condition, indicated that they were mainly affected by In/Directness and by the length of the stimulus (see Table 5, Fig. 1). Direct replies were responded to more quickly compared with indirect ones (*p* < 0.001, β = −0.012) and length of the target sentence also slowed down response times (*p* < 0.001, β = 0.019). There was no interaction between In/Directness and SA-matching (*p* = 0.192).

**Table 5:** Fixed and random effects for the model predicting log10(RT) data of the pragmatic task following sham TMS. Sum contrasts were used for all categorical predictors (see section 2.7).

RT (sham)					
log10 (RTs) ∼ in/directness* SA-matching + length + session + (1|subject) + (1|item)					
Fixed effects	β	Std. Error	df	z-value	p
Intercept	3.111	0.017	22.470	179.606	<0.001
In/Directness	−0.012	0.002	2567	−6.106	<0.001
SA-matching	0.001	0.004	123.800	0.236	0.814
Length	0.019	0.003	125.400	6.765	<0.001
Session	0.008	0.017	20.990	0.485	0.632
In/Directness: SA-matching	−0.003	0.002	2566	−1.306	0.192

Random effects	Variance	Std.Dev.			

Intercept (by subject)	0.001	0.036			
Intercept (by item)	0.007	0.081			
Residual	0.010	0.101

The analysis of the log-normalized RT data (sham and verum data together) in a larger model (see Table SB1) revealed an effect of In/Directness, so that reaction times to direct replies were shorter than to indirect ones (*p* < 0.001, β = −0.011). In addition, the length (in words) of the target utterance significantly increased the reaction times (*p* < 0.001, β = 0.020), whereas session also played a significant role, so that RTs were longer in the first than in the second session (*p* = 0.001, β = 0.015). A SA-matching by In/Directness interaction was also found (*p* = 0.01, β = −0.003, see Table SB2 for post-hoc tests). No further effects were found significant, particularly, no effect of Stimulation as a main effect or in interaction with other predictors.

**Fig. 2:**
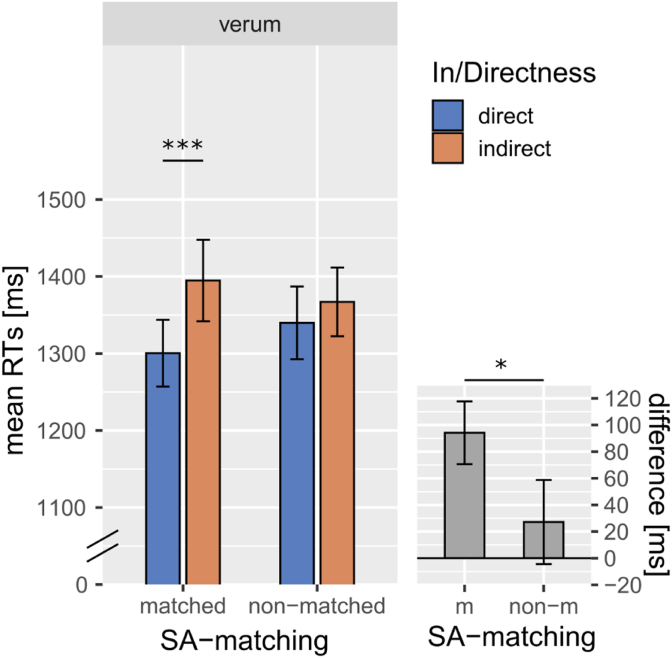
The large panel illustrates the average RTs (by subject) form the pragmatic task following verum TMS plotted by SA-matching and In/Directness, illustrating the significant interaction effect between In/Directness and SA-matching. The small panel illustrates the average difference in RTs found in the verum data between direct and indirect conditions (indirect>direct), separately by SA-matching. Error bars indicate the standard error of the mean (SEM) by subject. * indicates p < 0.05, ** indicates p < 0.01, *** indicates p < 0.001).

However, when analyzing the log-normalized RTs of the verum condition alone (see Table 6, Fig. 2), similar to what we did for the sham data, the same results of the sham analysis were reproduced (effect of In/Directness *p* < 0.001, β = −0.010; length of target sentence, *p* < 0.001, β = 0.022), with the difference that we found an additional significant interaction between In/Directness and SA-matching which was not present in the sham data (*p* = 0.014, β = −0.004). Follow up post-hoc tests indicated indeed that in the SA-matched set, indirect replies took longer to process than direct ones (*p*_Tukey_<0.001), however, this was not the case in the non-SA-matched set (*p*_Tukey_ = 0.167).

**Table 6:** Fixed and random effects for the model predicting log10(RT) data of the pragmatic task following verum TMS. Sum contrasts weres used for all categorical predictors (see section 2.7).

RT (verum)					
log10 (RTs) ∼ in/directness* SA-matching + length + session + (1|subject) + (1|item)					
Fixed effects	β	Std. Error	df	z-value	p
Intercept	3.110	0.014	26.740	219.043	<0.001
In/Directness	−0.010	0.002	2976	−5.453	<0.001
SA-matching	0.003	0.004	127	0.867	0.388
Length	0.022	0.003	128.400	7.924	<0.001
Session	0.021	0.014	23.990	1.529	0.139
In/Directness: SA-matching	−0.004	0.002	2976	−2.454	0.014

Random effects	Variance	Std.Dev			

Intercept (by subject)	0.001	0.038			
Intercept (by item)	0.005	0.070			
Residual	0.010	0.100

**Table 7:** Fixed and random effects for the model predicting log10(RT) data in the ToM task. Sum contrasts were used for all categorical predictors (see section 2.7).

RTs					
log10 (RTs) ∼ belief * desire * stimulation + length + session + (1|subject) + (1|item)					
Fixed effects	β	Std. Error	df	z-value	p
Intercept	2.829	0.014	25.260	200.893	<0.001
Belief	−0.052	0.002	5360	−27.998	<0.001
Desire	−0.046	0.002	5168	−24.505	<0.001
Stimulation	0.009	0.002	5377	4.924	<0.001
Session	0.014	0.002	5375	7.213	<0.001
Belief: Desire	−0.025	0.002	5362	−13.713	<0.001
Belief: Stimulation	−0.002	0.002	5359	−0.914	0.361
Desire: Stimulation	0.000	0.002	5362	−0.254	0.800
Belief: Desire: Stimulation	−0.003	0.002	5357	−1.674	0.094

Random effects	Variance	Std.Dev.			

Intercept (by subject)	0.005	0.071			
Intercept (by item)	0.000	0.004			
Residual	0.018	0.136

Analysis of the accuracy data did not reveal any significant effects of our factors of interest nor of the examined confound factors (see Table SA1).

### 3.2 Theory of mind task

In catch trials subjects achieved on average accuracy of 88.16% ± 0.09 SD, indicating that they paid attention to all information presented within each trial. The analysis of log-normalized RT data for the ToM task (see Table 7) indicated an effect of Belief so that true Belief was processed faster than false belief (*p* < 0.001, β = −0.052) and an effect of Desire (*p* < 0.001, β = −0.046) so that approach desire was processed faster than avoidance desire. In addition, there was a significant interaction between the two (*p* < 0.001, β = −0.025, see Table SD1 for post-hoc tests). Stimulation also had a significant facilitatory effect so that sham trials had longer RTs than verum trials (*p* < 0.001, β = 0.009). Interestingly, a marginally significant three-way interaction between Belief, Desire and Stimulation (*p* = 0.094, β = −0.003) was also detected. Follow-up post-hoc tests (see full Table SD2) on this three-way interaction indicated that while verum stimulation (vs. sham) did not affect the B+D+ conditions (*p*_Tukey_ = 0.950), it did decrease reaction times in the B + D- (*p* = 0.046) and in the B-D+ (*p*_Tukey_<0.01) but not in the B-D- condition, which did not survive correction for multiple comparisons (*p*_Tukey_ = 0.359, *p*_uncorrected_ = 0.029). The confound factor of Session also had a significant effect so that RTs were longer in the first than in the second session (*p* < 0.001, β = 0.014). Consistent with the analysis of RTs, the analysis of the accuracy showed an effect of Belief (*p* < 0.001, β = 0.682) and Desire (*p* < 0.001, β = 0.401), as well as an interaction between the two (*p* < 0.001, β = 0.201) and an effect of Session (*p* = 0.001, β = −0.152). However, it did not reveal any affect involving stimulation (see Table SC1 and SC.2).

## 4 Discussion

In the present study, we investigated whether activation in the right TPJ has a causal effect on the comprehension of direct and indirect speech acts. We tested this in two sets of conditions. In one set, direct and indirect speech acts were performed with the same utterance but differed in their communicative function (true question answering by a *statement* vs. *acceptance/declination* of an offer), in order to mimic a design used in most current studies. The other set of conditions was speech-act-matched and compared direct and indirect speech acts carried out with the same utterance and also with the same communicative role (true question answering by *stating*), therefore avoiding the confound of change in SA type. To obtain information about the participants’ speech act comprehension, a pragmatic task was applied: subjects had to decide whether the direct or indirect replies conveyed by identical sentences meant “yes” or “no” (that is, *agreement/disagreement* to a polar question or *acceptance/declination* of an offer/invitation). Following sham TMS (i.e., without manipulating brain activity) we found generally slowed pragmatic judgements for indirect compared with direct conditions regardless of SA-matching. This replicates established results and extends them to SA-matched indirect speech acts. However, following verum TMS of the right temporoparietal junction (rTPJ), pragmatic judgements were equally fast in non-SA-matched direct and indirect trials, whereas the in/directness contrast was significant for the conditions matched for speech act function (SA-matched). The observed response patterns show that, following TMS to the rTPJ, the well-established processing difference between direct and indirect speech acts is absent, but only if these are not matched for communicative function. As the processing difference between direct and indirect speech acts was still observed for items matched for communicative function, these results cannot be attributed to indirectness *per se*. In addition, we found some evidence that our TMS manipulation did affect ToM function as demonstrated by reduced RTs in a ToM task. Future studies need to investigate whether the observed pattern following verum TMS in the pragmatic task can be related to a difference in communicative function, e.g., *between agreement/disagreement* or *acceptance/declination*, or rather to a combination of speech act function and indirectness. Our data argue against a causal role of rTPJ in processing features specific to indirectness, such as the specific kind of ToM-related inferencing attributed to indirect communication.

### 4.1 Speech act type through the lenses of indirectness

Do the elevated processing costs of indirect speech acts relative to direct ones persist when both are matched for SA type? In conditions similar to natural ones, namely following sham TMS, subjects overall performed well and complied with task instruction across all experimental conditions as indicated by the average accuracy >90% (see Table SA1). This high accuracy is consistent with previous studies in which a task similar to ours was used and subjects had to indicate whether a given direct or indirect reply were interpreted as a “yes” or “no” (Feng et al., 2017, 2021; Jang et al., 2013). An absence of significant differences between direct and indirect conditions was also previously reported in two studies by Feng et al. (2017, 2021). In contrast, Jang et al. (2013), who also employed the same pragmatic task, did find indirect replies to be understood with significantly less accuracy compared with direct ones. In their task however, they did not use the same linguistic form across direct and indirect conditions. Their finding of different accuracy for direct and indirect replies might therefore be related to an absence of structural matching of the direct/indirect stimuli. Therefore, our results are consistent with those of other studies (Feng et al., 2017, 2021) that used carefully matched direct and indirect stimuli consisting of identical sentences in the direct and indirect condition, in spite of the absence of matching of direct and indirect conditions for the type of speech act used. Concerning reaction times following sham TMS (Fig. 1), in our study we find that subjects were slower at responding to indirect vs. direct replies. We therefore replicate the consistent finding that indirect replies take longer to process than direct ones. Such a difference in response times was previously identified in a range of tasks including reading times (Hamblin and Gibbs, 2003; Holtgraves, 1999), attribution of a “negative, positive or meaningless” connotation (Shibata et al., 2011) and yes/no interpretation of the indirect response (Feng et al., 2017, 2021; Jang et al., 2013), which was also applied here. However, this previous literature did not examine the potential role of SA-changes co-occurring with indirectness. Instead, they typically examined either indirect speech acts involving SA change or a mixture of indirect replies with and without SA-change. Crucially, we presently extend these findings by demonstrating that these processing delays are maintained, also when the indirect reply does not involve a change of SA function relative to its direct control. Therefore, it appears that processing indirectness requires additional cognitive processing regardless of SA-matching. It is possible that these additional processes are involved in the inference that is required to identify the indirectly conveyed meaning. However, a previous study has highlighted that indirect speech acts are typically less predictable, less semantically similar to their context question, less coherent with their context question and understood with less certainty compared with direct ones conveyed by the same utterance (Boux et al., 2023). Each of these features and any combination between them, as well as differential ToM involvement, could be at the basis of a relatively higher difficulty or load in processing indirect speech acts, as indexed by prolonged reaction times. The exact cause of such processing differences however cannot be revealed by reaction times data alone and is discussed in the next section.

Does the rTPJ play a causal role in indirect speech acts (vs. direct ones) with or without speech act matching? For the reaction times obtained following verum TMS, a two-way interaction emerged between In/Directness and SA-matching that was not present in the absence of TMS (i.e., *sham* condition). Indeed, when the rTPJ was stimulated with TMS, the difference in reaction times between the indirect and direct replies was preserved in the SA-matched set, whereas a corresponding difference for the non-matched set was not reliable (Fig. 2). This can be seen as evidence that, following TMS to the rTPJ, the well-known effect of indirectness on response times is not present if the direct and indirect conditions are not matched for speech act function. However, with matching for speech act type, the indirectness difference is still present, similar to the results in the sham condition where magnetic stimulation is ineffective. As there is an interaction effect following TMS, which was not detected following sham TMS, these data suggest that TMS to rTPJ changes the normally seen patterns only in case indirectness is accompanied by a change in communicative function. Therefore, these results can be used to argue for a functional relevance of rTPJ for SA-nonmatched in/directness, but not for indirectness, because SA-matching is the only feature discriminating between conditions. One may question the above interpretation by pointing to the larger statistical analysis involving both *sham* and *verum* conditions together. In this case, the three-way interaction between in/Directness, SA-matching and Stimulation did not reach significance. This is a limitation, given that such a significant three-way interaction would have provided the strongest evidence for a differential effect of TMS on direct and indirect SA-matching and –mismatching communicative actions. It is possible that the sample size of the present study was not large enough to achieve sufficient statistical power for obtaining a significant 3-way interaction effect. Note that, in the absence of openly available pre-existing data on this topic, a power-analysis during study planning was not possible. Similarly, the difference between RTs to non-SA-matched direct and indirect replies following verum TMS was not statistically significant with *p*_*Tukey*_ = 0.167. Here again, we cannot exclude that this difference would have been significant with a larger sample size.

However, the pattern of results obtained provides some indication regarding the role of rTPJ in comprehension of indirectness. We do not find evidence that rTPJ is *causally* involved in comprehension of indirectness *per se*. If this were the case, the reaction time differences between direct and indirect replies would not have been detectable any longer neither in the SA-matched nor in the non-SA-matched set following verum stimulation. Instead, we only find evidence that, when rTPJ was stimulated, the comprehension of the indirect replies in the non-SA-matched set was affected. Therefore, whether indirectness co-occurred with a change in speech act seems to be a key element in explaining our results. For this reason, we suggest that, following rTPJ stimulation, SA-matched indirect replies behaved in the same way as documented in the literature and in our present sham experiment, but that, following verum TMS, non-SA-matched ones failed to show this difference normally reported. In the absence of a significant triple interaction, we interpret this as some, although moderate, evidence for a role of the rTPJ in contributing to the processing difference between not-SA—matched direct and indirect speech acts.

### 4.2 Theory of mind

**Fig. 3:**
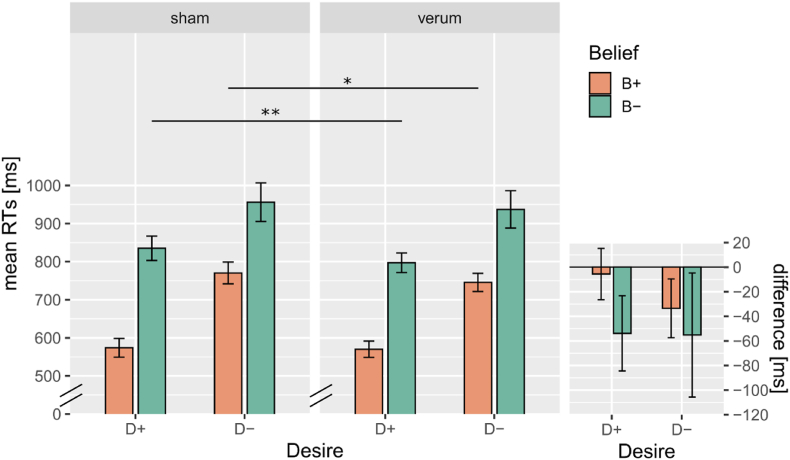
The large panel illustrates the reaction time data form the Theory of Mind task separated by Belief and Desire, where B+: true belief, B-: false belief, D+: approach desire and D-: avoidance desire. The small panel illustrated the difference in reaction times between verum and sham condition (verum>sham) by Belief and Desire. In all panels, error bars indicate the standard error of the mean (SEM) by subject. Stars indicate significance level (* indicates p < 0.05, ** indicates p < 0.01, *** indicates p < 0.001).

The ToM task captured two classic components of Theory of Mind, i.e., processing of (true/false) beliefs and processing of (approach/avoidance) desires (Leslie et al., 2004; Premack and Woodruff, 1978; Wellman and Liu, 2004). As expected, false belief trials (B-) were more difficult to process than true belief trials (B+) and avoidance desire (D-) trials were more difficult to process than the approach trials (D+). This was manifest in the form of longer reaction times and lower accuracies. Furthermore, we found an interaction effect between belief and desire, so that trials combining a false belief and an aversive desire (B-D-) were the longest to process, but were nevertheless processed faster than a mere additive effect of Belief and Desire would predict. So, overall, we successfully replicated the known effects associated with this specific task and other variants thereof (Apperly et al., 2011; Hartwright et al., 2012, 2014, 2016) indicating that these two aspects of ToM, namely processing of beliefs and of desires, require a cognitive effort. Importantly, this also indicates that our task was effectively implemented. In addition, we find several indicators that TMS successfully affected ToM processing. Indeed, a marginally significant effect of stimulation was detected in the reaction times measures. Importantly, stimulation decreased reaction times in ToM trials involving avoidance desire (B + D-) and false belief (B-D+) but not in the non-ToM control condition (B + D+), which remained unaffected. Despite a numeric difference, the effect of stimulation on the ToM condition involving both avoidance desire and false belief (B-D-) was not significant after correction for multiple comparison. It is possible that the combined ToM condition did not only require higher engagement of ToM, but also of other processes (e.g., increased attention). This could potentially have made the data more variable (note indeed larger error bars for the B+D- conditions in Fig. 3), which in turn could have made the TMS effect more difficult to detect, resulting in only a marginal significance.

### 4.3 Stimulation of the rTPJ

An important aspect of our results that needs to be addressed is the directionality of the effects. By “directionality”, we mean whether the TMS manipulation resulted in facilitation or inhibition and thus in speeding or slowing of behavioral responses. Regarding the pragmatic task, it was surprising, on first view, that the reaction time difference between SA-matched indirect and direct replies with TMS to the rTPJ was smaller than in the non-SA-matched set. As the alteration relates only to the RT *differences* between direct and indirect speech acts, it is not possible to talk with certainty about a facilitation, although the pattern of result can be seen to be compatible with a facilitation effect. In contrast, verum TMS significantly reduced reaction times in the critical trials of the ToM task therefore showing behavioral facilitation. In fact, the stimulation protocol used in the present study (i.e., 1 Hz TMS pulses for 25 min) is expected to produce inhibition in the targeted brain area (Fitzgerald et al., 2006; Silvanto and Cattaneo, 2017) and previous studies directing TMS to the rTPJ consistently found that 1 Hz off-line TMS protocol produced inhibitory effects on ToM function or on other aspects of social cognition (Baumgartner et al., 2014; Costa et al., 2008; Giardina et al., 2011; Young et al., 2010). In this research context, our present results appear as a case of “paradoxical facilitation”, i.e., the detection of behavioral facilitation in an inhibitory TMS protocol, which is sometimes observed in non-invasive brain stimulation research (Najib and Pascual-Leone, 2011; Théoret et al., 2003). Of course, such labeling does not provide an explanation for why the reverse effects are present in our study, nevertheless, paradoxical facilitation still indicates that TMS targeting the rTPJ had a causal role in the affected conditions. One might hypothesize that our TMS manipulation induced a speed accuracy tradeoff. However, this interpretation is not supported by the data because there were no differences in accuracy in either the pragmatic or the ToM task, although RTs suggested facilitation in both tasks.

Another concern might relate to the possibility of the stimulation effects spreading from our stimulation target to other brain areas. TMS is known to alter function not only in the target brain area, but also in adjacent areas and in connected ones, including the homotopic areas in the hemisphere contralateral to the stimulation (Bergmann and Hartwigsen, 2020). Therefore, one could suspect that our TMS to the rTPJ might have caused alterations in the functionality of the left TPJ, a region contributing to semantic processing (Binder et al., 2009). Indirect replies are indeed more semantically distant from their context than their direct counterparts, but this is unaffected by SA-matching (see Boux et al., 2023 who tested a nearly identical stimulus set). Therefore, if TMS had affected semantic processing, one would have expected that both SA-matched and non-SA-matched indirect replies to be equally altered following verum TMS. However, our pattern of results is inconsistent with this possibility, as we only find RTs to non-SA-matched indirect speech acts to remain different from direct ones following verum TMS. We therefore find it unlikely that our results can be explained by TMS affecting the left TPJ.

### 4.4 Speech acts, indirect speech acts and theory of mind

Are alterations in reaction times to non-SA-matched indirect replies observed following verum TMS to the rTPJ related to ToM processing? In the pragmatic task, we used a well-matched stimulus set (see Table 2, Table 3) where direct and indirect replies were conveyed by the very same linguistic form and we applied a within subjects experimental design. In addition, we find some parallels between TMS-associated alterations of performance in the pragmatic task and TMS-induced alterations in the ToM task. These are consistent with the involvement of ToM in comprehension of non-SA-matched indirect speech acts (but not of matched ones).

How can this different pattern of results be explained? The rTPJ's role in processing the non-SA-matched set may in part be due to the specific *types* of communicative actions performed by the indirect replies, that is, to their illocutionary roles (Searle, 1979). By definition, ToM includes the processing of shared assumptions, beliefs and intentions of the communication partners, which are relevant for understanding a communicative action. For instance, healthy adults are known to keep track of the state of knowledge of other people during conversation (Rueschemeyer et al., 2015). In case of non-SA-matched indirect replies, a closer assessment of the common ground between the conversational partners might be required to understand or infer the communicative function of accepting or rejecting an offer/invitation compared with an assertive speech act and therefore result in a greater ToM load. In sum, it is possible that non-SA-matched indirect replies included indirect speech acts that are particularly reliant on ToM and that were therefore sensitive to TMS to rTPJ, whereas SA-matched ones did not.

In the light of these considerations and of the findings of the present study, we suggest a reinterpretation of the past literature. As already argued in the introduction, much of the past research did not systematically take SA-matching as a relevant factor in their experimental design. In fact, most or even all of the stimuli used were indirect speech acts that co-occurred with SA-change. (We write “most”, because, for some studies, the methods descriptions do not include sufficient information about speech act type). No previous study reported to have performed the SA matching we argue is necessary to unconfound studies of indirectness. Interestingly, these studies find direct associations between indirect language processing and ToM either directly, by behavioral correlations (Champagne-Lavau and Joanette, 2009; Champagne-Lavau and Stip, 2010; Trott and Bergen, 2018), or indirectly, by finding the ToM brain network to be active during comprehension of indirectness (Bašnáková et al., 2014, 2015; Feng et al., 2017, 2021; Jang et al., 2013; Shibata et al., 2011; van Ackeren et al., 2012). A neurostimulation study targeting rTPJ using tDCS also found evidence consistent with a causal role of this region for comprehension of indirect speech acts, via a modulation of ToM function (Feng et al., 2021).

The present finding that SA-matching might play a role in the involvement of ToM is compatible with the findings of these previous studies, and potentially offers a different interpretative key. Namely, it appears possible that at least some of the ToM activation found in the previous studies could have been in fact due to contrasting different speech act types at the direct and indirect level. In other words, these might reflect differences in processing of the speech act types rather than differences in processing indirectness *per se*. This view becomes particularly plausible if one considers the difference between speech acts such as *statements* on the one hand and other speech acts such as *excuses*, *promises*, *negative opinions*, etc. on the other. If someone *states* that China is far away, this communication may involve little ToM processing. However, if one uses the same sentence as an *excuse* to justify the postponement of e.g., an important business trip, some evaluation of possible partner responses, including thoughts and plans, seems likely. Therefore, it is evident that different communicative actions, even entirely direct ones, come with different ToM load.

When looking at the broader picture of non-literal language comprehension, two positions emerge. One position sees ToM as having a central role in non-literal language processing in general (Sperber and Wilson, 1995, 2002). The other postulates that different non-literal language phenomena might rely on ToM to different degrees, so that some non-literal phenomena might possibly not require additional ToM (Andrés-Roqueta and Katsos, 2017; Bosco et al., 2018; Domaneschi and Bambini, 2020; Jary, 2013; Kissine, 2016). Our present findings are compatible with the rTPJ being relevant specifically for comprehension of indirect speech acts co-occurring with a speech act change relative to their direct controls. We also find that the rTPJ might contribute to the comprehension by supporting ToM function. An involvement of rTPJ-mediated ToM in comprehension of indirectness *per se* would have been demonstrated only by finding an effect of TMS on behavior both in the SA-matched and non-SA-matched studies. Indeed, the SA-matched set was the only condition that had indirectness fully isolated from other factors such as SA-change. As, following TMS stimulation, these well-matched conditions failed to yield differences between direct and indirect communication, the results do not support a specific role of the stimulated area in indirectness. The significant difference seen in the non-SA-matched conditions is congruent with the assumption that the difference in speech act function was relevant for the TMS effect, but is equally compatible with rTPJ-mediated ToM being causally involved when in/directness and communicative function change occur jointly. In a scenario in which the non-SA-matched indirect speech acts depend on ToM even more than SA-matched ones, the TMS manipulation might have affected only the more “ToM greedy” condition but not the SA-matched ones. To sum up, although we cannot speak to the larger question of a specific involvement of ToM in indirect speech acts, we do find support for the necessity of rTPJ-mediated ToM when speech act function and in/directness status are both altered.

So, we know that speech act matching has an effect on the cognitive (Boux et al., 2023) and, as suggested by the present data, on neuronal processes underlying comprehension of indirectness. We also know that different (direct) speech act types have different neural signatures, sometimes involving substantially different sets of cortical areas (Boux et al., 2021; Egorova et al., 2013, 2014, 2016; Tomasello et al., 2019, 2022; van Ackeren et al., 2012, 2016). Therefore, it seems that the so far common experimental approach of comparing direct speech acts used to carry out a certain communicative function with indirect ones that are used to realize a different communication function might not be the best experimental approach. This type of contrast might, together with the neurocognitive basis of indirectness, also capture a functional SA difference, which can act as a confound factor. We suggest that future research should be conducted in awareness of this confound. Ideally, SA-type should be controlled for by examining direct and indirect speech acts carrying out the same type of communicative action. Alternatively, our present approach could also be taken, having different sets of SA-matched and SA-unmatched direct and indirect stimuli. In addition, specificity and transparency of the methods section regarding the types of SAs carried out directly and indirectly would be desirable to facilitate comparability between studies and could be achieved by having these reported systematically. Finally, our present results strongly suggest investigating in greater detail the different common ground and theory of mind processes characterizing different communicative functions along with their cortical activation signatures.

### 4.5 Conclusion

In the present study, we asked whether previously reported findings about processing differences between direct and indirect speech acts can indeed be attributed to in/directness. A review of the literature shows that, in most or all previous work, the in/directness difference was confounded by the use of different speech act types in the direct and indirect conditions (e.g., *statements*vs. *requests*). As brain imaging studies showed that brain activation changes with communicative function, we here compared conditions in which the speech act performed directly and indirectly were not matched for speech act type, as in the previous work, with novel conditions in which direct and indirect communicative actions were performed with the same sentence and speech act function. The findings are as follows: (1) following sham TMS (i.e., in the absence of actual brain stimulation), we replicate that indirect speech acts take longer to process than matched direct controls for non-SA-matched conditions and extend this finding to SA-matched ones. (2) Following verum TMS of the right temporal junction, rTPJ, a brain site thought to be relevant for Theory of Mind processing, the response time difference between non-SA-matched indirect and direct communicative actions is absent, which is consistent with a role of the stimulated cortical region in indirect SA processing. (3) However, there was no comparable pattern following verum TMS when direct and indirect conditions were matched for speech act function. SA-matched direct and indirect conditions showed the same significant response time difference regardless of TMS manipulation. This result argues against the possibility of the rTPJ being important for indirectness processing *per se*. (4) The TMS manipulation facilitated processing in the critical trials in a ToM task, a finding consistent with a role of this area in ToM processing. We conclude that the rTPJ is causally involved in indirect (vs. direct) speech act processing, but only if an additional difference in speech act function is present. Therefore, the role of this region is not specific or indicative of indirectness *per se*. Our results suggest that activation of ToM systems found in previous neuroimaging studies for comprehension of indirectness might likewise be, at least in part, due to co-occurring SA changes. Our work comes with the methodological implications for future studies of indirectness that it is essential to match not only for critical linguistic structures – the words and sentences used as tools to perform direct or indirect communicative actions - but to match, in addition, for the type of speech act, as different speech acts come with different requirements on calculating the knowledge and commitments of communication partners. Furthermore, we suggest exploring in detail the different ToM requirements of specific communicative functions along with their correlated and causal brain loci.

## Author contributions

Isabella Boux: Conceptualization, Methodology, Formal analysis, Investigation, Data Curation, Writing - Original Draft and Review & Editing, Visualization, Project administration; Friedemann Pulvermüller: Conceptualization, Methodology, Resources, Writing – Original Draft and Review & Editing, Supervision, Funding acquisition, Project administration.

## Declaration of competing interest

The authors declare no conflict of interest.

## Data Availability

The authors do not have permission to share raw data. The study materials for the pragmatic task were made available at https://osf.io/myhc7/. The Theory of Mind Task was taken from Hartwright et al. (2012). We therefore refer those who would like to obtain the material for this task to the original creators.
