# Supplementary material for: Does the right temporo-parietal junction play a role in processing indirect speech acts? A transcranial magnetic stimulation study
Source: Neuropsychologia. 2023 Sep 9;188:108588. doi: 10.1016/j.neuropsychologia.2023.108588 (PMC10498423; doi:10.1016/j.neuropsychologia.2023.108588)
Supplement: Multimedia component 1 [file mmc1.docx]

**Supplementary Material A**

*Accuracy at the Pragmatic Task*

As the hypothesized model (P) failed to converge for the accuracy data, a simplified model was tested, dropping the confound predictors (Pa), which however also did not converge. Thus, our final model was (Pc) with the confound predictors included, but with the random term for by-item intercepts dropped.

(P) Variable ~ In/Directness * Stimulation * SA-matching + length + session + (1|subject) + (1|item)

(Pa) Variable ~ In/Directness * Stimulation * SA-matching + (1|subject) + (1|item)

(Pb) Variable ~ In/Directness * Stimulation * SA-matching + length + session + (1|subject)

| **ACCURACY** | | | | |
| --- | --- | --- | --- | --- |
| **accuracy ~ in/directness * SA-matching * stimulation + length + session + (1\|subject)** | | | | |
| **Fixed effects** | **β** | **Std. Error** | **z-value** | **p** |
| Intercept | 2.913 | 0.118 | 24.705 | <0.001 |
| In/Directness | -0.019 | 0.053 | -0.363 | 0.717 |
| SA-matching | -0.089 | 0.053 | -1.674 | 0.094 |
| Stimulation | -0.077 | 0.054 | -1.421 | 0.155 |
| Length | -0.138 | 0.038 | -3.607 | <0.001 |
| Session | -0.135 | 0.054 | -2.498 | 0.012 |
| In/Directness : SA-matching | 0.006 | 0.053 | 0.109 | 0.913 |
| In/Directness : Stimulation | -0.062 | 0.053 | -1.166 | 0.244 |
| SA-matching : Stimulation | -0.054 | 0.053 | -1.013 | 0.311 |
| In/Directness : SA-matching : Stimulation | -0.059 | 0.053 | -1.116 | 0.264 |
| **Random effects** | **Variance** | **Std.Dev.** |  |  |
| Intercept (by subject) | 0.2611 | 0.511 |  |  |

Table SA.1: *Fixed and random effects for the model predicting accuracy data in the Pragmatic task. Sum contrast was used for all categorical predictors (see section 2.7).*

**Supplementary Material B**

*RTs at the Pragmatic Task*

| **RTs** | | | | | |
| --- | --- | --- | --- | --- | --- |
| **log10(RTs) ~ in/directness* SA-matching * stimulation + length + session + (1\|subject) + (1\|item)** | | | | | |
| **Fixed effects** | **β** | **Std. Error** | **df** | **z-value** | **p** |
| Intercept | 3.111 | 0.014 | 27.890 | 220.067 | <0.001 |
| In/Directness | -0.011 | 0.001 | 5678 | -7.942 | <0.001 |
| SA-matching | 0.002 | 0.004 | 126.600 | 0.582 | 0.562 |
| Stimulation | 0.001 | 0.001 | 5685 | 0.425 | 0.671 |
| Length | 0.020 | 0.003 | 127.200 | 7.946 | <0.001 |
| Session | 0.015 | 0.001 | 5685 | 10.637 | <0.001 |
| In/Directness : SA-matching | -0.003 | 0.001 | 5678 | -2.569 | 0.010 |
| In/Directness : Stimulation | -0.001 | 0.001 | 5677 | -0.841 | 0.401 |
| SA-matching : Stimulation | -0.001 | 0.001 | 5675 | -0.709 | 0.478 |
| In/Directness : SA-matching : Stimulation | 0.001 | 0.001 | 5677 | 0.837 | 0.403 |
| **Random effects** | **Variance** | **Std.Dev.** |  |  |  |
| Intercept (by subject) | 0.001 | 0.037 |  |  |  |
| Intercept (by item) | 0.005 | 0.070 |  |  |  |
| Residual | 0.010 | 0.102 |  |  |  |

*Table SB.1: Fixed and random effects for the model predicting RT data in the Pragmatic task. Sum contrast was used for all categorical predictors (see section 2.7).*

| **contrast** | | | **estimate** | **SE** | **df** | **z.ratio** | **p_uncorrected_** | **p_Tukey_** |
| --- | --- | --- | --- | --- | --- | --- | --- | --- |
| direct/SA-matched | - | indirect/SA-matched | -0.028 | 0.004 | 5682 | -7.643 | <.0001 | <.0001 |
| direct/SA-matched | - | direct/non-SA-matched | -0.003 | 0.008 | 169 | -0.366 | 0.715 | 0.983 |
| direct/SA-matched | - | indirect/non-SA-matched | -0.017 | 0.008 | 169 | -2.268 | 0.025 | 0.110 |
| indirect/SA-matched | - | direct/non-SA-matched | 0.025 | 0.008 | 168 | 3.358 | 0.001 | 0.005 |
| indirect/SA-matched | - | indirect/non-SA-matched | 0.011 | 0.008 | 169 | 1.454 | 0.148 | 0.468 |
| direct/non-SA-matched | - | indirect/non-SA-matched | -0.014 | 0.004 | 5679 | -3.700 | 0.000 | 0.001 |

*Table SB.2: Post-hoc tests on to further elucidate the lack of significant of the In/Directness * SA-matching interaction in the sham condition. Results are averaged over the levels of stimulation and session. The Kenward-Roger was used for calculating degrees of freedom. P-values are corrected for a family of 4 estimates by Tukey’s HSD method. Relevant comparisons are highlighted in grey.*

| **contrast** | | | **estimate** | **SE** | **df** | **z.ratio** | **p_uncorrected_** | **p_Tukey_** |
| --- | --- | --- | --- | --- | --- | --- | --- | --- |
| direct/SA-matched | - | indirect/SA-matched | -0.028 | 0.005 | 2977 | -5.762 | <.0001 | <.0001 |
| direct/SA-matched | - | direct/non-SA-matched | -0.002 | 0.008 | 194 | -0.273 | 0.785 | 0.993 |
| direct/SA-matched | - | indirect/non-SA-matched | -0.013 | 0.008 | 193 | -1.564 | 0.119 | 0.402 |
| indirect/SA-matched | - | direct/non-SA-matched | 0.026 | 0.008 | 194 | 3.125 | 0.002 | 0.011 |
| indirect/SA-matched | - | indirect/non-SA-matched | 0.015 | 0.008 | 193 | 1.839 | 0.067 | 0.258 |
| direct/non-SA-matched | - | indirect/non-SA-matched | -0.011 | 0.005 | 2979 | -2.060 | 0.040 | 0.167 |

*Table SB.3: Post-hoc tests on to further elucidate the significant of the In/Directness * SA-matching interaction in the verum condition. Results are averaged over the levels of stimulation and session. The Kenward-Roger was used for calculating degrees of freedom. P-values are corrected for a family of 4 estimates by Tukey’s HSD method. Relevant comparisons are highlighted in grey.*

**Supplementary Material C**

*Accuracy in the Theory of Mind Task*

| **ACCURACY** |  |  |  |  |
| --- | --- | --- | --- | --- |
| **accuracy ~ belief * desire * stimulation + length + session + (1\|subject) + (1\|item)** | | | | |
| **Fixed effects** | **β** | **Std. Error** | **z-value** | **p** |
| Intercept | 2.520 | 0.153 | 16.516 | <0.001 |
| Belief | 0.682 | 0.053 | 12.995 | <0.001 |
| Desire | 0.401 | 0.053 | 7.636 | <0.001 |
| Stimulation | -0.020 | 0.054 | -0.364 | 0.715 |
| Session | -0.152 | 0.045 | -3.382 | 0.001 |
| Belief : Desire | 0.201 | 0.052 | 3.832 | <0.001 |
| Belief : Stimulation | 0.024 | 0.052 | 0.463 | 0.643 |
| Desire : Stimulation | -0.014 | 0.052 | -0.261 | 0.794 |
| Belief : Desire : Stimulation | -0.057 | 0.052 | -1.082 | 0.279 |
| **Random effects** | **Variance** | **Std.Dev.** |  |  |
| Intercept (by subject) | 0.488 | 0.698 |  |  |
| Intercept (by item) | 0.013 | 0.114 |  |  |

*Table SC.1: Fixed and random effects for the model predicting accuracy data in the ToM task. Sum contrast was used for all categorical predictors (see section 2.7).*

| **contrast** | | | **estimate** | **SE** | **df** | **z.ratio** | **p_uncorrected_** | **p_Tukey_** |
| --- | --- | --- | --- | --- | --- | --- | --- | --- |
| B+/D+ | - | B-/D+ | 1.766 | 0.175 | Inf | 10.112 | <.0001 | <.0001 |
| B+/D+ | - | B+/D- | 1.203 | 0.183 | Inf | 6.573 | <.0001 | <.0001 |
| B+/D+ | - | B-/D- | 2.166 | 0.171 | Inf | 12.65 | <.0001 | <.0001 |
| B-/D+ | - | B+/D- | -0.563 | 0.122 | Inf | -4.629 | <.0001 | <.0001 |
| B-/D+ | - | B-/D- | 0.4 | 0.102 | Inf | 3.908 | 0.0001 | 0.0005 |
| B+/D- | - | B-/D- | 0.963 | 0.116 | Inf | 8.289 | <.0001 | <.0001 |

*Table SC.2: Post-hoc tests on to further elucidate the marginally significant Belief * Desire interaction. Results are averaged over the levels of stimulation and session. The Kenward-Roger was used for calculating degrees of freedom. P-values are corrected for a family of 4 estimates by Tukey’s HSD method. Relevant comparisons are highlighted in grey.*

**Supplementary Material D**

*RTs in the Theory of Mind Task*

| **contrast** | | | **estimate** | **SE** | **df** | **z.ratio** | **p_uncorrected_** | **p_Tukey_** |
| --- | --- | --- | --- | --- | --- | --- | --- | --- |
| B+/D+ | - | B-/D+ | -0.155 | 0.005 | 5362.000 | -29.759 | <.0001 | <.0001 |
| B+/D+ | - | B+/D- | -0.142 | 0.005 | 5285.000 | -27.634 | <.0001 | <.0001 |
| B+/D+ | - | B-/D- | -0.195 | 0.005 | 5319.000 | -36.995 | <.0001 | <.0001 |
| B-/D+ | - | B+/D- | 0.013 | 0.005 | 5330.000 | 2.461 | 0.014 | 0.066 |
| B-/D+ | - | B-/D- | -0.040 | 0.005 | 5335.000 | -7.478 | <.0001 | <.0001 |
| B+/D- | - | B-/D- | -0.053 | 0.005 | 5361.000 | -10.025 | <.0001 | <.0001 |

*Table SD.1: Post-hoc tests on to further elucidate the marginally significant Belief * Stimulation interaction. Results are averaged over the levels of Session and Desire. The Kenward-Roger was used for calculating degrees of freedom. P-values are corrected for a family of 4 estimates by Tukey’s HSD method. Relevant comparisons are highlighted in grey.*

| **contrast** | | | **estimate** | **SE** | **df** | **z.ratio** | **p_uncorrected_** | **p_Tukey_** |
| --- | --- | --- | --- | --- | --- | --- | --- | --- |
| B+/D+/sham | - | B-/D+/sham | -0.165 | 0.008 | 5361 | -21.688 | <.0001 | <.0001 |
| B+/D+/sham | - | B+/D-/sham | -0.149 | 0.007 | 5331 | -20.002 | <.0001 | <.0001 |
| B+/D+/sham | - | B-/D-/sham | -0.199 | 0.008 | 5321 | -25.919 | <.0001 | <.0001 |
| B+/D+/sham | - | B+/D+/verum | 0.008 | 0.007 | 5367 | 1.133 | 0.257 | 0.950 |
| B+/D+/sham | - | B-/D+/verum | -0.137 | 0.007 | 5366 | -18.518 | <.0001 | <.0001 |
| B+/D+/sham | - | B+/D-/verum | -0.127 | 0.007 | 5355 | -17.213 | <.0001 | <.0001 |
| B+/D+/sham | - | B-/D-/verum | -0.183 | 0.007 | 5367 | -24.399 | <.0001 | <.0001 |
| B-/D+/sham | - | B+/D-/sham | 0.015 | 0.008 | 5338 | 2.019 | 0.044 | 0.469 |
| B-/D+/sham | - | B-/D-/sham | -0.035 | 0.008 | 5328 | -4.457 | <.0001 | 0.0002 |
| B-/D+/sham | - | B+/D+/verum | 0.173 | 0.007 | 5368 | 23.285 | <.0001 | <.0001 |
| B-/D+/sham | - | B-/D+/verum | 0.027 | 0.008 | 5364 | 3.633 | 0.000 | 0.007 |
| B-/D+/sham | - | B+/D-/verum | 0.038 | 0.008 | 5366 | 5.049 | <.0001 | <.0001 |
| B-/D+/sham | - | B-/D-/verum | -0.018 | 0.008 | 5366 | -2.366 | 0.018 | 0.258 |
| B+/D-/sham | - | B-/D-/sham | -0.050 | 0.008 | 5360 | -6.538 | <.0001 | <.0001 |
| B+/D-/sham | - | B+/D+/verum | 0.157 | 0.007 | 5353 | 21.607 | <.0001 | <.0001 |
| B+/D-/sham | - | B-/D+/verum | 0.012 | 0.007 | 5362 | 1.627 | 0.104 | 0.734 |
| B+/D-/sham | - | B+/D-/verum | 0.023 | 0.007 | 5366 | 3.063 | 0.002 | 0.046 |
| B+/D-/sham | - | B-/D-/verum | -0.033 | 0.007 | 5367 | -4.46 | <.0001 | 0.0002 |
| B-/D-/sham | - | B+/D+/verum | 0.208 | 0.008 | 5348 | 27.6 | <.0001 | <.0001 |
| B-/D-/sham | - | B-/D+/verum | 0.062 | 0.008 | 5360 | 8.147 | <.0001 | <.0001 |
| B-/D-/sham | - | B+/D-/verum | 0.073 | 0.008 | 5367 | 9.589 | <.0001 | <.0001 |
| B-/D-/sham | - | B-/D-/verum | 0.017 | 0.008 | 5366 | 2.188 | 0.029 | 0.359 |
| B+/D+/verum | - | B-/D+/verum | -0.145 | 0.007 | 5360 | -20.392 | <.0001 | <.0001 |
| B+/D+/verum | - | B+/D-/verum | -0.135 | 0.007 | 5361 | -19.12 | <.0001 | <.0001 |
| B+/D+/verum | - | B-/D-/verum | -0.191 | 0.007 | 5361 | -26.518 | <.0001 | <.0001 |
| B-/D+/verum | - | B+/D-/verum | 0.010 | 0.007 | 5362 | 1.451 | 0.147 | 0.833 |
| B-/D+/verum | - | B-/D-/verum | -0.045 | 0.007 | 5360 | -6.186 | <.0001 | <.0001 |
| B+/D-/verum | - | B-/D-/verum | -0.056 | 0.007 | 5360 | -7.681 | <.0001 | <.0001 |

*Table SD.2: Post-hoc tests on to further elucidate the marginally significant Belief * Desire * Stimulation interaction. Results are averaged over the levels of Session. The Kenward-Roger was used for calculating degrees of freedom. P-values are corrected for a family of 8 estimates by Tukey’s HSD method. Relevant comparisons are highlighted in grey.*
